# Differential loss to follow-up rates among adult tuberculosis patients – Findings from the largest private sector tuberculosis clinic database in Myanmar

**DOI:** 10.1371/journal.pone.0218450

**Published:** 2019-06-17

**Authors:** Ye Kyaw Aung, Phyu Phyu Swe, Zayar Kyaw, Si Thu Thein

**Affiliations:** Population Services International, Yangon, Myanmar; Saint Louis University, UNITED STATES

## Abstract

**Background:**

Population Services International (PSI) Myanmar’s social franchise network of general practitioners, known as Sun Quality Health Clinics (SQHC), provided tuberculosis (TB) diagnosis and treatment with Direct Observed Treatment Short course (DOTs) across Myanmar since 2004, with a total of 898 clinics across the country. People who sought TB treatment from these SQHC by themselves were regarded as walk-in patients. To augment TB case notification, PSI Myanmar developed two treatment seeking channels: Community Health Services Providers (CHSP) and Interpersonal Communicators (IPC). They actively sought people who were suspected to have TB and referred them to SQH clinics. In this study, we compared the loss to follow-up rates of TB patients across three treatment seeking channels; and investigated risk-factors for loss to follow-up.

**Methods and findings:**

A retrospective cohort design was applied using TB client records between 2012 and 2016. Outcome was defined as loss to follow-up in comparison to successful TB treatment (completed or cured). Multivariate Poisson regression was conducted to estimate incidence rate ratio of loss to follow-up. Of the 62,664 TB patients registered at the SQHC, around 10% each were actively screened by the CHSP and the IPC, and 78.9% were walk-in patients. Overall cumulative incidence for loss to follow-up rate was significantly higher in the IPC channel (14.2%, 95% CI 13.4–15.1%) than walk-in patients (8.9%, 95% CI 8.6–9.1%) and the CHSP channel (5.5%, 95% CI 5.0–6.1%) (p<0.001). The median time after which patients were lost to follow-up from treatment was 4.04 months. We found that patients with older age, male sex, patients residing in hilly region, unknown smear status, retreated cases, HIV co-infection, and unknown HIV status were risk-factors for loss to follow-up in the continuation phase of treatment; whereas patients with higher initial body weight, patients who received travel support and patients taking treatment in older providers were less likely to be lost to follow-up.

**Conclusions:**

Based on these findings, we recommend that implementation strategies for improving case notification and treatment seeking should carefully consider retention strategies in parallel, and the identified influencing factors for loss to follow-up should be taken account for such consideration.

## Introduction

Tuberculosis (TB) remains as one of the major causes of illness and death worldwide. Myanmar is listed as one of the world’s 30 high TB burden countries [1], with the incidence rate of 361 cases per 100,000 population, compared to the global average of 140 cases per 100,000 population in 2016 [2]. World Health Organization (WHO) reported that Myanmar experienced a consistent decline in the prevalence, incidence and TB death rates from 1990 to 2014 [3]. At the same times, TB notification rates have increased sharply since the 1990s, when an average of 20,000 cases were notified per year [4]. In 2014, a total of 141,957 cases were notified across the country, 82% by the National TB program and 18% by private practitioners, non-governmental organizations (NGO) and hospitals [4].

Directly observed treatment short course (DOTS) is the most effective mean of eliminating TB from a population. It needs to be taken at least 6 months to complete a full course. In those TB patients required to undergo lengthy treatment, poor adherence or loss to follow-up is a common problem. Failure to complete treatment or cure is found to be the main reason for difficulties in controlling any kind of disease including TB. At the national level in Myanmar, treatment success rates of 85% have been achieved in 2008 with 4.2% loss to follow-up rate [5]. The treatment success rates in Myanmar has been increased to 87% and 85% in 2013 and 2014 cohort, but with 5% and 5.2% loss to follow-up rate, respectively [2, 4]. Though treatment success rate has not been changed much in later years, it still has a higher loss to follow-up rate than that of the previous year.

The previous quantitative data from the international studies identified several reasons and risk factors for unsuccessful treatment outcomes including loss to follow-up [6–13]. Male patients, HIV positivity, smear-negative pulmonary TB and extra-pulmonary TB were most often found to be risk factors for unsuccessful treatment outcomes including loss to follow-up [6–13]. Additionally, the patients living in communities far from the treatment center [13], lack of contact person [11], smear positive result at 2 or 3 months after initial treatment [9, 11], advanced age [6, 10], unemployment [10], multidrug-resistant TB [10], malignancy [10], previous default [8], alcohol abuse [8], and so on contributed to loss to follow-up from TB treatment.

In Myanmar, a study conducted in the Yangon and Mandalay Regions showed that up to 30% of the drug-resistant TB treatment cases experienced initial loss to follow-up, which was significantly higher among older patients [14]. However, the studies regarding loss to follow-up among all TB patients after initiating treatment are scarce in Myanmar. To our knowledge, there were no comparison studies of loss to follow-up rates among different treatment seeking channels in Myanmar.

Population Services International (PSI) Myanmar’s public-private mix for DOTS (PPM-DOTS) clinics are franchised network of general practitioner clinics named as the Sun Quality Health Clinics (SQHC), providing TB diagnosis and treatment with DOTS across Myanmar since 2004. Up to 2016, there were 898 SQHC clinics under PPM-DOTS program in Myanmar. TB patients were registered at the SQHC via three different treatment seeking channels, which were, directly to the SQHC as walk-in patients, interpersonal communicators (IPC) and community health services providers (CHSP). Different treatment seeking channels have applied different approaches for finding of presumptive case finding although the SQHC have provided the same standardized TB treatment according to the National TB Program guidelines.

At the SQHC, the providers passively diagnosed TB among patients who came to the clinic by themselves (walk-in patients). Most of those patients lived in vicinity of clinics and they usually had close relationship with the providers.

Since 2008, community health services providers (CHSP) channel had been launched and community volunteers were trained for providing package of community based TB care (CBTBC) services which included active TB case finding and referral. They mainly worked in rural area and linked rural population to the nearest SQHC. In addition to case finding and referral, the CHSP supervised regular drug taking to ensure TB treatment adherence (DOTS supervision) and provided other related services like contact tracing, sputum transportation for diagnosis and follow-up recheck and drug issue on behalf of patients in need.

Interpersonal communicator (IPC) model started in 2011, with an aim to reach urban slum population who usually did not come to clinics unless they are seriously ill. The IPC, who were the trained PSI staff, actively sought the presumptive TB cases in the target population through health talk activities. They referred presumptive TB cases for sputum examination, provided sputum transportation service in necessary area, and chest X-ray. After receiving investigation results, they accompanied the patients to consult with SQHC doctors for further management. As their main responsibility was active detection of TB cases and effective referral to reach nearest SQHC, the IPC did not involve in treatment adherence support once TB patients got registered at clinic.

Based on the routinely reported data of PSI-Myanmar’s PPM-DOTS clinics, the loss to follow-up rate had been increasing with the rate of 6.8%, 8.1%, 8.6%, in 2012, 2013 and 2014, respectively. The rates declined slightly in 2015 and 2016 to 8.2% and 7.8%, respectively. However, the rates were higher than 5% loss to follow-up rate for Myanmar in the Global Tuberculosis report 2015 [4]. Therefore, this study aimed to investigate overall cumulative incidence of loss to follow-up from TB treatment and factors which may influence loss to follow-up, by using the register data of SQHC clinics, segregated across the different treatment seeking channels.

## Methods

### Study design

A retrospective cohort study design was applied using the data from TB client register of PSI/Myanmar’s 898 PPM-DOTS clinics between 2012 and 2016.

### Study population

The study population were all adult TB patients, who were registered at the SQHC clinics for DOTS from January 2012 to December 2016 with the treatment outcomes of ‘cured’, ‘treatment completed’ and ‘loss to follow-up’. All the patients included in the study were regarded as TB patients only if their sputum result showed smear-positive, their chest X’ray showed pulmonary TB and/or other diagnosis tools showed TB confirmed, for example, TB meningitis in cerebrospinal fluid examination. Those patients with other outcomes: died, treatment failure, transferred-out and moved to second line drugs, were excluded from the study. The ‘cured’ and ‘treatment completed’ outcomes were operationally defined as ‘treatment success’ [15]. In this study, the ‘cured’ was defined as those TB patients who completed their respective treatment and their sputum result at the fifth month of the treatment showed ‘smear-negative’. The ‘treatment completed’ meant those TB patients who completed their respective treatment, but not performed the sputum test at the fifth month of the treatment. The outcome of loss to follow-up was defined as a TB patient whose treatment was interrupted for two consecutive months or more [15].

### Data extraction

The data were extracted from the electronic registers, which included clients’ basic information: unique identification number, sex, age, body weight at diagnosis, address, patient type (new, relapse, failure and defaulted) disease site (pulmonary, primary complex and extra-pulmonary), sputum results, date of treatment started, treatment regime (initial regimen, retreatment regimen), travel support, nutritional support, HIV test result, treatment seeking channels (walk-in, community health services provider and interpersonal communicator), treatment outcomes (cured, completed, died, failure, loss to follow-up, transferred out and moved to second line drugs) and date of treatment outcomes. Additionally, the basic information of SQHC providers was extracted from the list of SQH provider database, which included age, sex, and years in experience of working with DOTS.

### Statistical analysis

Data analyses were performed using STATA 14.2 for window version [16]. Distribution of continuous variables were checked by histogram and categorized into groups if not normally distributed. Years of provider experience with DOTS were calculated by subtracting the last date of treatment outcomes (31^st^ December 2016) from the date of joining TB program. The treatment time for individuals was calculated from the treatment started date until treatment completion or loss to follow-up. Treatment month was defined as 28 days a month. Kaplan-Meier estimator was used to analyze the time of loss to follow-up from treatment and to draw cumulative event curves. Cumulative incidence for loss to follow-up was estimated among the adult TB patients of treatment success and loss to follow-up, for three different phases (the first two months of treatment, the first 6 month of treatment and overall).

The incidence rate of loss to follow-up varied across the treatment phases, namely the intensive phase for the first two months of treatment, the continuation phase for up to six months of treatment, and the continuation phase beyond the six months of treatment. In the initial analysis, we fitted Cox proportional hazard regression model to identify the influencing factors. However, we found that the hazard ratios for loss to follow-up were not proportional across several factors, and Cox model was not appropriate for our case.

Therefore, we used multivariate Poisson regression model to compare the incidence rates of loss to follow-up during each phase of treatment and identify influencing factors. For Poisson regression models, the main outcome measure of comparison was incidence rate ratio (IRR), defined as the ratio of the incidence rates between comparison groups. Univariate and multivariate Poisson regression models were fitted using IRR of loss to follow-up as outcome measure, and potential influencing factors for loss to follow-up as independent variables in each phase of treatment. Two-sided significant tests with p-values of <0.05 were considered statistically significant.

### Ethical considerations

The study protocol was approved by the Ethics Review Committee of the Department of Medical Research, Ministry of Health and Sports, Myanmar. Personally identifiable information of TB patients and DOTS providers were not extracted from the registers to protect their confidentiality.

## Results

### Characteristics of patients

The study population comprised of a total of 62,664 adult TB patients were registered at the SQHC clinics during the study period (Table 1). Of those, 62% were male and 38% were female. The patients were of an average age of 43 years and an average initial body weight of 45.2 kg. The majority of patients resided in Delta region (50.3%) and Plain Region (34.4%), and only 6.5% in Hilly Region. The majority (97.4%) were pulmonary TB cases and only 2.6% were extra-pulmonary TB cases. The sputum smears were positive in 50.6%, negative in 47.7%, and not available in 1.7%. Most of the patients (93.4%) were new cases and only 6.6% were retreated ones. Among the patients, 3% were known HIV positive, 50.2% known HIV negative, and 46.9% did not know their HIV status.

**Table 1:** Characteristics of adult tuberculosis patients across three different treatment seeking channels.

Characteristics	All participants (N = 62,664)		Walk-in Channel (N = 49,453)			Community health services provider (CHSP) Channel (N = 6,380)		Interpersonal Communicator (IPC) Channel (N = 6,831)	
	N	%/SD	N	%/SD		N	%/SD	N	%/SD
**Demographic characteristics**									
**Age (years)**									
Average	43.0	16.3	42.6	16.4		44.8	15.6	43.8	15.7
**Sex**									
Male	38,848	62.0%	30,840	62.4%		3,812	59.7%	4,196	61.4%
Female	23,816	38.0%	18,613	37.6%		2,568	40.3%	2,635	38.6%
**Initial body weight**									
Average	45.2	8.8	45.3	8.8		44.0	8.2	45.3	9.2
**Region**									
Delta region	31,547	50.3%	22,834	46.2%		3,328	52.2%	5,385	78.8%
Hilly region	4,093	6.5%	3,905	7.9%		182	2.9%	6	0.1%
Plain region	21,549	34.4%	18,403	37.2%		1,707	26.8%	1,439	21.1%
Coastal region	5,475	8.7%	4,311	8.7%		1,163	18.2%	1	0.0%
**Clinical characteristics**									
**Disease status**									
Pulmonary	61,020	97.4%	47,909	96.9%		6,315	99.0%	6,796	99.5%
Extra-pulmonary	1,644	2.6%	1,544	3.1%		65	1.0%	35	0.5%
**Sputum result**									
Smear positive	31,679	50.6%	24,936	50.4%		3,075	48.2%	3,668	53.7%
Smear negative	29,889	47.7%	23,514	47.5%		3,251	51.0%	3,124	45.7%
Not done	1,096	1.7%	1,003	2.0%		54	0.8%	39	0.6%
**Types of cases**							0.0%		
New	58,543	93.4%	46,102	93.2%		6,092	95.5%	6,349	92.9%
Retreated	4,121	6.6%	3,351	6.8%		288	4.5%	482	7.1%
**Status of HIV**									
Negative	31,434	50.2%	24,253	49.0%		3,581	56.1%	3,600	52.7%
Positive	1,871	3.0%	1,615	3.3%		98	1.5%	158	2.3%
Unknown	29,359	46.9%	23,585	47.7%		2,701	42.3%	3,073	45.0%
**Support characteristics**									
**Travel support**									
Yes	8,834	14.1%	5,848	11.8%		2,221	34.8%	765	11.2%
No	53,830	85.9%	43,605	88.2%		4,159	65.2%	6,066	88.8%
**Nutrition support**									
Yes	21,438	34.2%	16,647	33.7%		2,660	41.7%	2,131	31.2%
No	41,226	65.8%	32,806	66.3%		3,720	58.3%	4,700	68.8%
**Treatment starting year**									
2012	13,474	21.5%	11,433	23.1%		1,099	17.2%	942	13.8%
2013	14,730	23.5%	11,166	22.6%		1,568	24.6%	1,996	29.2%
2014	13,042	20.8%	10,402	21.0%		1,315	20.6%	1,325	19.4%
2015	11,329	18.1%	9,055	18.3%		1,053	16.5%	1,221	17.9%
2016	10,089	16.1%	7,397	15.0%		1,345	21.1%	1,347	19.7%
**Service providers' characteristics**									
**Age (years)**									
26–40	6,220	9.9%	5,095	10.3%		700	11.0%	425	6.2%
40+	44,697	71.3%	35,100	71.0%		4,793	75.1%	4,804	70.3%
Unknown	11,747	18.7%	9,258	18.7%		887	13.9%	1,602	23.5%
**Sex**									
Male	45,251	72.2%	36,673	74.2%		4,230	66.3%	4,348	63.7%
Female	17,350	27.7%	12,717	25.7%		2,150	33.7%	2,483	36.3%
Health facility	63	0.1%	63	0.1%		-		-	
**Experience with TB program (years)**									
0–5	4,990	8.0%	4,272	8.6%		348	5.5%	370	5.4%
6–10	29,537	47.1%	21,542	43.6%		4,767	74.7%	3,228	47.3%
10+	28,137	44.9%	23,639	47.8%		1,265	19.8%	3,233	47.3%

N: Number; SD: Standard Deviation; Delta region includes Yangon and Ayeyarwaddy Division; Hilly region includes Chin, Kachin, Kayah, Kayin, Shan East, Shan North and Shan South regions; Plain region includes Bago East, Bago West, Magway, Mandalay, Naypyitaw and Sagaing; Coastal region includes Mon, Tanintharyi and Rakhine regions; Retreated cases means all patient types with previously treatment.

Regarding the program perspectives, PSI supported for 14.1% of the patients for their travel to respective treatment centers, and 34.2% for their nutrition. DOTS provision for the majority of the patients were from providers over 40 years of age (71.3%), predominantly males (72.2%), with at least 6 years of experience in TB program (91.4%).

Regarding the initial treatment seeking channels, 78.9% were walk-in patients, 10.2% from CHSP and 10.9% from IPC. The general characteristics of patients were similar across these three channels (Table 1).

### Cumulative incidence of loss to follow-up

**Fig 1:**
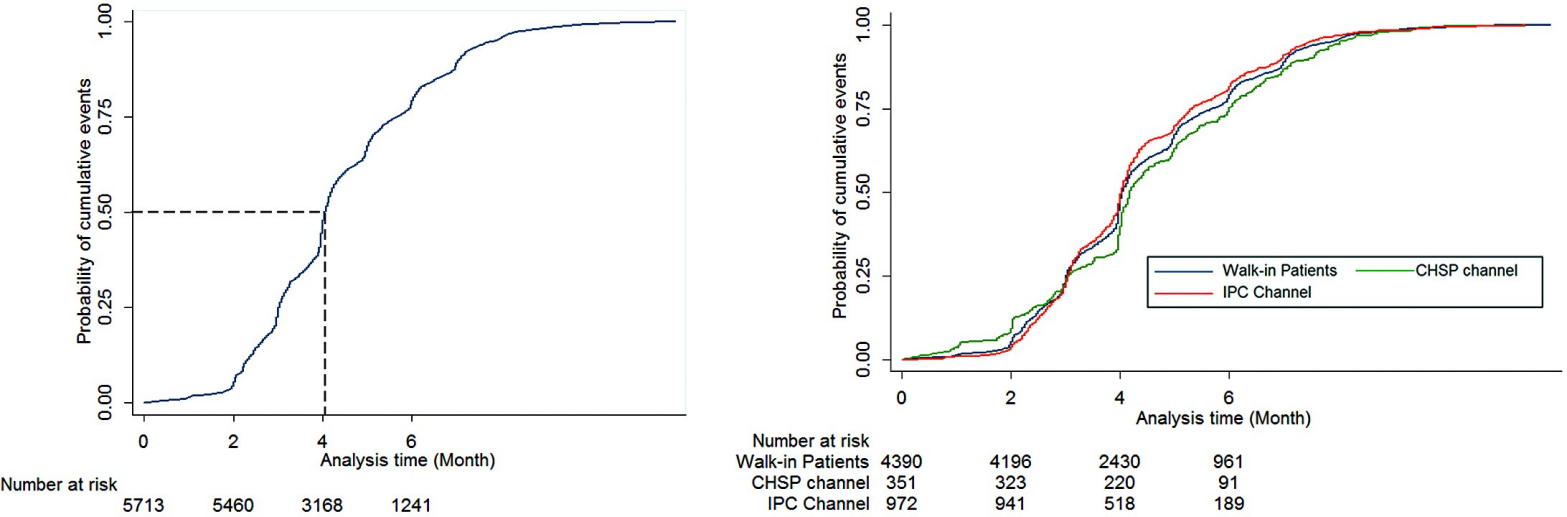
Cumulative event graph of loss to follow-up patients.

Fig 1 illustrated the cumulative events of patients who were lost to follow-up over the treatment duration across different channels. The overall median time after which the patients were lost to follow-up was 4.04 months. The loss to follow-up rate rose sharply after the initial phase and plateaued after six months. The patterns of loss to follow-up were different across the three treatment seeking channels.

Hence, we analyzed the cumulative incidence of loss to follow-up for each channel for each treatment phase (Table 2). The overall cumulative incidence of loss to follow-up was 9.1% (95% CI, 8.9% - 9.3%). Across three treatment seeking channels, the overall cumulative incidence was the highest among the patients referred from the IPC channel, (14.2%, 95% CI 13.4% - 15.1%), followed by the walk-in patients (8.9%, 95% CI 8.6% - 9.1%), and the patients referred from CHSP channel (5.5%, 95% CI 5.0% - 6.1%).

**Table 2:** Cumulative incidence* of loss to follow-up across treatment phases and across different treatment seeking channels.

Characteristics	Cumulative incidence during the first 2 months (intensive phase)**	Cumulative incidence during the first 6 months**	Overall cumulative incidence**	Overall cumulative incidence across different channels % (95% CI)		
	% (95% CI)	% (95% CI)	% (95% CI)	Walk-in channel	CHSP channel	IPC channel
Overall	0.5% (0.4% - 0.6%)	7.2% (7.0% - 7.5%)	9.1% (8.9% - 9.3%)	8.9% (8.6% - 9.1%)	5.5% (5.0% - 6.1%)	14.2% (13.4% - 15.1%)
**Treatment seeking channels**						
Walk-in	0.5% (0.4% - 0.6%)	7.0% (6.8% - 7.3%)	8.9% (8.6% - 9.1%)	-	-	-
Community health services provider (CHSP)	0.5% (0.4% - 0.7%)	4.2% (3.7% - 4.7%)	5.5% (5.0% - 6.1%)	-	-	-
Interpersonal communicator (IPC)	0.6% (0.4% - 0.8%)	11.6% (10.9% - 12.4%)	14.2% (13.4% - 15.1%)	-	-	-
**Demographic characteristics**						
**Sex**						
Male	0.5% (0.5% - 0.6%)	7.8% (7.5% - 8.0%)	10.0% (9.7% - 10.3%)	9.8% (9.5% - 10.2%)	5.9% (5.2% - 6.7%)	15.2% (14.1% - 16.3%)
Female	0.4% (0.4% - 0.5%)	6.4% (6.1% - 6.7%)	7.6% (7.3% - 8.0%)	7.3% (6.9% - 7.7%)	4.9% (4.1% - 5.8%)	12.7% (11.5% - 14.1%)
**Region**						
Delta region	0.5% (0.4% - 0.5%)	7.7% (7.4% - 8.0%)	9.8% (9.5% - 10.1%)	9.0% (8.6% - 9.3%)	6.0% (5.2% - 6.8%)	15.7% (14.7% - 16.7%)
Hilly region	0.3% (0.2% - 0.5%)	12.9% (11.9% - 14.0%)	16.2% (15.1% - 17.4%)	16.7% (15.5% - 17.9%)	7.1% (3.9% - 11.9%)	-
Plain region	0.5% (0.4% - 0.6%)	6.2% (5.9% - 6.5%)	7.6% (7.3% - 8.0%)	7.8% (7.4% - 8.2%)	4.3% (3.4% - 5.4%)	8.8% (7.3% - 10.3%)
Coastal region	1.0% (0.8% - 1.3%)	4.7% (4.2% - 5.3%)	5.8% (5.2% - 6.5%)	5.9% (5.2% - 6.6%)	5.6% (4.3% - 7.1%)	-
**Clinical characteristics **						
**Disease status**						
Pulmonary	0.5% (0.4% - 0.6%)	7.2% (7.0% - 7.4%)	9.1% (8.9% - 9.3%)	8.9% (8.6% - 9.1%)	5.5% (5.0% - 6.1%)	14.2% (13.4% - 15.1%)
Extra-pulmonary	0.5% (0.2% - 1.0%)	7.8% (6.5% - 9.2%)	9.1% (7.8% - 10.6%)	9.3% (7.9% - 10.8%)	4.6% (1.0% - 12.9%)	11.4% (3.2% - 26.7%)
**Sputum result**						
Smear positive	0.4% (0.3% - 0.5%)	6.5% (6.2% - 6.8%)	9.0% (8.7% - 9.3%)	8.4% (8.0% - 8.7%)	5.7% (4.9% - 6.5%)	15.9% (14.7% - 17.1%)
Smear negative	0.6% (0.5% - 0.7%)	7.9% (7.6% - 8.2%)	9.1% (8.8% - 9.4%)	9.2% (8.8% - 9.6%)	5.3% (4.6% - 6.1%)	12.2% (11.0% - 13.4%)
Not done	1.0% (0.5% - 1.8%)	12.0% (10.2% - 14.1%)	14.1% (12.0% - 16.3%)	14.2% (12.1% - 16.5%)	7.4% (2.1% - 17.9%)	20.5% (9.3% - 36.5%)
**Types of cases**						
New	0.5% (0.5% - 0.6%)	7.2% (7.0% - 7.4%)	8.9% (8.7% - 9.2%)	8.7% (8.5% - 9.0%)	5.4% (4.9% - 6.0%)	13.9% (13.1% - 14.8%)
Retreated	0.3% (0.1% - 0.5%)	8.1% (7.2% - 8.9%)	11.8% (10.8% - 12.8%)	11.2% (10.2% - 12.3%)	6.9% (4.3% - 10.5%)	18.5% (15.1% - 22.2%)
**Status of HIV**						
Negative	0.5% (0.4% - 0.6%)	5.4% (5.1% - 5.6%)	6.9% (6.6% - 7.1%)	6.7% (6.4% - 7.0%)	4.9% (4.3% - 5.7%)	9.9% (9.0% - 10.9%)
Positive	0.6% (0.3% - 1.0%)	8.7% (7.4% - 10.0%)	10.8% (9.5% - 12.3%)	10.5% (9.1% - 12.1%)	14.3% (8.0% - 22.8%)	12.0% (7.4% - 18.1%)
Unknown	0.5% (0.4% - 0.6%)	9.2% (8.8% - 9.5%)	11.4% (11.1% - 11.8%)	11.0% (10.6% - 11.4%)	5.9% (5.1% - 6.9%)	19.4% (18.0% - 20.8%)
**Support characteristics**						
**Travel support**						
Yes	0.3% (0.2% - 0.5%)	3.7% (3.3% - 4.1%)	5.0% (4.6% - 5.5%)	4.6% (4.1% - 5.2%)	3.8% (3.1% - 4.7%)	11.6% (9.4% - 14.1%)
No	0.5% (0.5% - 0.6%)	7.8% (7.6% - 8.1%)	9.8% (9.5% - 10.0%)	9.5% (9.2% - 9.7%)	6.4% (5.7% - 7.2%)	14.6% (13.7% - 15.5%)
**Nutrition support**						
Yes	0.5% (0.5% - 0.7%)	6.6% (6.3% - 7.0%)	8.6% (8.2% - 9.0%)	8.2% (7.8% - 8.6%)	5.9% (5.1% - 6.9%)	15.1% (13.6% - 16.6%)
No	0.5% (0.4% - 0.6%)	7.6% (7.3% - 7.8%)	9.4% (9.1% - 9.7%)	9.2% (8.9% - 9.5%)	5.2% (4.5% - 6.0%)	13.9% (12.9% - 14.9%)
**Treatment starting year**						
2012	0.1% (0.1% - 0.2%)	6.5% (6.1% - 7.0%)	8.2% (7.8% - 8.7%)	7.8% (7.3% - 8.3%)	5.0% (3.8% - 6.5%)	17.4% (15.0% - 20.0%)
2013	0.6% (0.5% - 0.8%)	7.6% (7.2% - 8.0%)	9.2% (8.8% - 9.7%)	8.6% (8.0% - 9.1%)	4.6% (3.6% - 5.7%)	16.7% (15.1% - 18.4%)
2014	0.6% (0.4% - 0.7%)	8.1% (7.6% - 8.6%)	10.2% (9.7% - 10.7%)	10.0% (9.5% - 10.6%)	6.8% (5.5% - 8.3%)	14.6% (12.8% - 16.7%)
2015	0.6% (0.4% - 0.7%)	7.3% (6.8% - 7.8%)	9.3% (8.8% - 9.9%)	9.4% (8.8% - 10.0%)	4.9% (3.7% - 6.4%)	12.8% (11.0% - 14.9%)
2016	0.7% (0.5% - 0.8%)	6.6% (6.1% - 7.1%)	8.5% (8.0% - 9.1%)	8.8% (8.2% - 9.5%)	6.2% (4.9% - 7.6%)	9.1% (7.6% - 10.8%)
**Service providers' characteristics**						
**Age (years)**						
26–40	0.6% (0.4% - 0.8%)	8.0% (7.3% - 8.7%)	10.4% (9.6% - 11.2%)	10.4% (9.6% - 11.3%)	4.9% (3.4% - 6.7%)	19.1% (15.4% - 23.1%)
40+	0.5% (0.4% - 0.5%)	6.5% (6.3% - 6.7%)	8.2% (8.0% - 8.5%)	8.0% (7.8% - 8.3%)	4.8% (4.2% - 5.4%)	12.9% (12.0% - 13.9%)
Unknown	0.6% (0.4% - 0.7%)	9.7% (9.2% - 10.3%)	11.9% (11.3% - 12.5%)	11.2% (10.6% - 11.9%)	9.9% (8.0% - 12.1%)	16.9% (15.1% - 18.8%)
**Sex**						
Male	0.6% (0.5% - 0.6%)	7.1% (6.9% - 7.4%)	8.9% (8.6% - 9.2%)	8.7% (8.4% - 9.0%)	5.0% (4.4% - 5.7%)	14.4% (13.4% - 15.5%)
Female	0.3% (0.3% - 0.4%)	7.5% (7.1% - 7.9%)	9.7% (9.2% - 10.1%)	9.4% (8.9% - 9.9%)	6.4% (5.4% - 7.5%)	13.9% (12.6% - 15.4%)
Health facility	3.2% (0.4% - 11.0%)	9.5% (3.6% - 19.6%)	11.1% (4.6% - 21.6%)	11.1% (4.6% - 21.6%)	-	-
**Experience with TB program (years)**						
0–5	0.7% (0.5% - 0.9%)	9.6% (8.8% - 10.5%)	12.7% (11.8% - 13.6%)	12.4% (11.4% - 13.4%)	7.2% (4.7% - 10.5%)	21.3% (17.2% - 25.8%)
6–10	0.6% (0.5% - 0.7%)	7.0% (6.7% - 7.3%)	8.7% (8.4% - 9.0%)	8.9% (8.5% - 9.3%)	5.6% (4.9% - 6.2%)	12.4% (11.3% - 13.6%)
10+	0.4% (0.3% - 0.5%)	7.1% (6.8% - 7.4%)	8.9% (8.6% - 9.2%)	8.3% (7.9% - 8.6%)	4.8% (3.7% - 6.2%)	15.2% (14.0% - 16.5%)

*Cumulative incidence for loss to follow-up was calculated after excluding other outcomes (died, failure, moved to second line and transferred out), and therefore the estimates were relatively higher;

**the loss to follow-up events during the first two months, during the first six months and overall were 315, 4542, and 5713, respectively;

CI: Confidence Interval.

When looking over treatment phases, the overall cumulative incidence was only 0.5% (95% CI, 0.5% - 0.6%) during the intensive phase, but rose to 7.2% (95% CI 7.0% - 7.5%) during the continuation phase (up to 6 months). Similar patterns were seen across all three treatment seeking channels.

Regarding patients’ characteristics, overall cumulative incidence was higher in male patients (10%, 95% CI 9.7% - 10.3%) than in females (7.6%, 95% CI 7.3% - 8%). Geographically, the overall cumulative incidence was the highest among patients from hilly regions (16.2%, 95% CI 15.1% - 17.4%), followed by those from delta regions (9.8%, 95% CI 9.5% - 10.1%), plain region (7.6%, 95% CI 7.3% - 8%), and the coastal regions (5.8%, 95% CI 5.2% - 6.5%). In contrast, similar overall cumulative incidence was seen among pulmonary and extra-pulmonary TB patients, (9.1%, 95% CI 8.9% - 9.3%) vs. (9.1%, 95% CI 7.8% - 10.6%). Also, the overall cumulative incidence did not differ between sputum smear-positive patients (9.0%, 95% CI 8.7% - 9.3%) and sputum smear-negative ones (9.1%, 95% CI 8.8% - 9.4%), but those with no sputum results had higher overall cumulative incidence (14.1%, 95% CI 12.0% - 16.3%). Retreated patients had higher overall cumulative incidence (11.8%, 95% CI 10.8% - 12.8%) than the newly identified patients (8.9%, 95% CI 8.7% - 9.2%). Likewise, the overall cumulative incidence was higher in known HIV positive patients (10.8%, 95% CI 9.5% - 12.3%) and those with unknown HIV status (11.4%, 95% CI 11.1% - 11.8%), compared to known HIV-negative TB patients (6.9%, 95% CI 6.6% - 7.1%).

Regarding the program support activities, the patients who received travel support had lower overall cumulative incidence (5.0%, 95% CI 4.6% - 5.5%) compared to those who did not (9.8%, 95% CI 9.5% - 10%), whereas similar overall cumulative incidence was seen among those patients with and without nutrition support (8.6%, 95% CI 8.2% - 9.0% vs. 9.4%, 95% CI 9.1% - 9.7%). Chronologically from 2012 to 2016, overall cumulative incidence fluctuated between the lowest of 8.2% to the highest of 10.2%.

In regards to SQHC providers, the overall cumulative incidence was lower in the patients from the providers over 40 years of age (8.2%, 95% CI 8.0% - 8.5%), compared to those from younger providers (10.4%, 95% CI 9.6% - 11.2%). Also, overall cumulative incidence was slightly lower in the patients from the male providers (8.9%, 95% CI 8.6% - 9.2%) than those from the female providers (9.7%, 95% CI 9.2% - 10.1%). In contrast, the overall cumulative incidence was the highest among patients from the providers with less experience in TB program (5-year and below) (12.7%, 95% CI 11.8% - 13.6%), compared to other groups.

As described in Table 2, similar patterns of the variations in overall cumulative incidence were seen across all three treatment seeking channels.

### Risk factors for loss to follow-up

**Fig 2:**
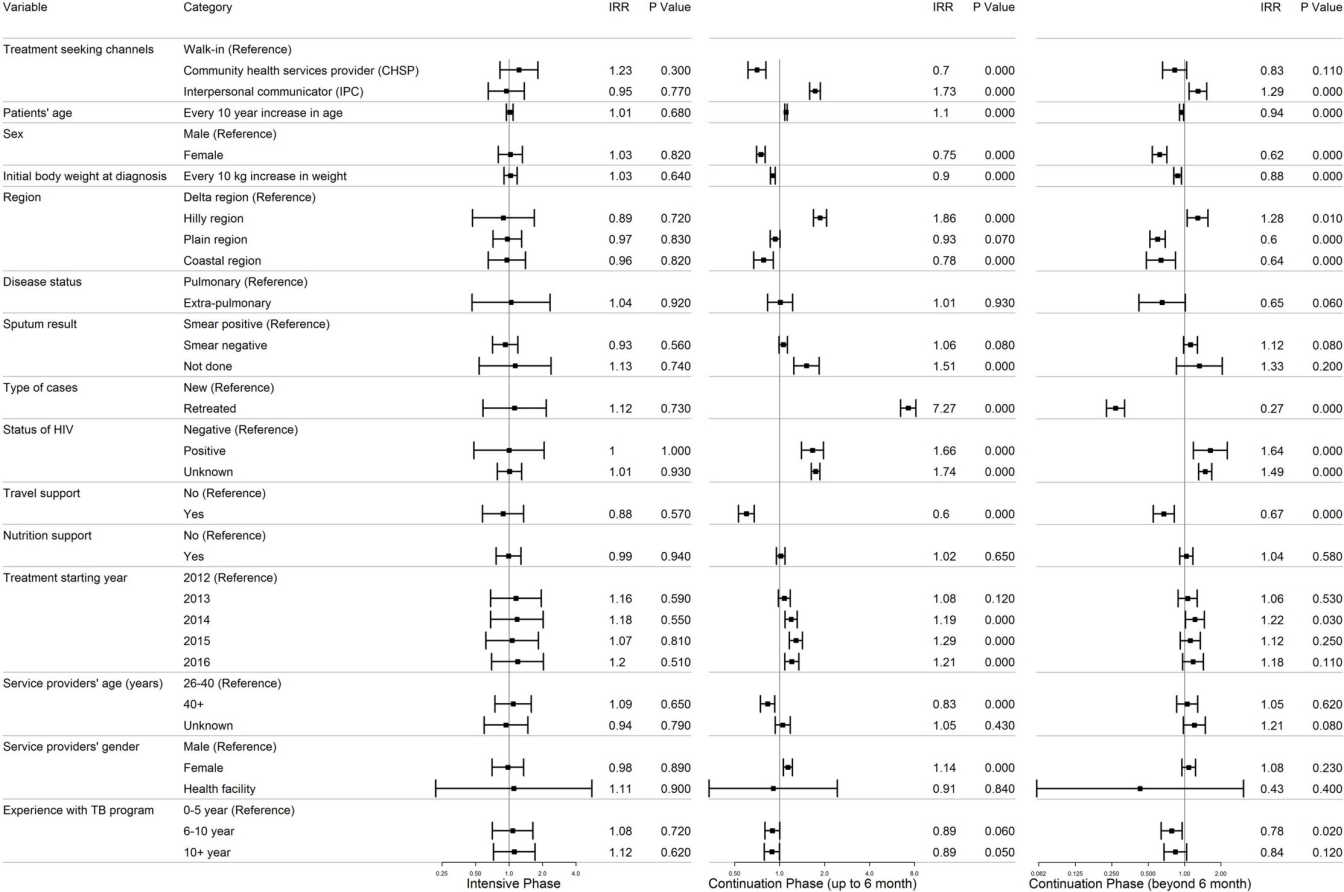
Multivariate Poisson regression results of factors related to loss to follow-up over different treatment phases.

Table 3 showed the risk factors for loss to follow-up, using incidence rate ratios (IRR) from univariate and multivariate Poisson regression models, across different treatment phases. During the intensive phase (first two months), there was no difference in adjusted IRR among any factors, including the treatment seeking channels. However, both continuation phases (up to 6 months, and beyond 6 months) showed variations in IRR across several factors. The results of multivariate Poisson regression over different treatment phases were summarized in Fig 2.

**Table 3:** Factors related to loss to follow-up rates across different treatment phases.

Characteristics	Intensive phase						Continuation phase						Continuation phase beyond 6 month					
	Unadjusted			Adjusted			Unadjusted			Adjusted			Unadjusted			Adjusted		
	IRR	95% CI	p-value*	IRR	95% CI	p-value**	IRR	95% CI	p-value*	IRR	95% CI	p-value**	IRR	95% CI	p-value*	IRR	95% CI	p-value**
**Treatment seeking channels**																		
**Walk-in**	**Reference**			**Reference**			**Reference**			**Reference**			**Reference**			**Reference**		
**Community health services provider (CHSP)**	1.21	0.84–1.74	0.304	1.23	0.83–1.82	0.304	0.58	0.51–0.67	<0.001	0.7	0.61–0.81	<0.001	0.8	0.64–0.99	0.043	0.83	0.66–1.04	0.109
**Interpersonal communicator (IPC)**	0.95	0.68–1.34	0.779	0.95	0.65–1.37	0.771	1.72	1.59–1.87	<0.001	1.73	1.59–1.88	<0.001	1.44	1.22–1.69	<0.001	1.29	1.09–1.53	0.003
**Demographic characteristic**																		
**Age (years)**																		
	**Reference**			**Reference**			**Reference**			**Reference**			**Reference**			**Reference**		
**Every 10 year increase in age**^**a**^	1.01	0.95–1.08	0.686	1.01	0.95–1.09	0.684	1.12	1.1–1.14	<0.001	1.1	1.08–1.12	<0.001	0.91	0.87–0.94	<0.001	0.94	0.91–0.98	0.003
**Sex**																		
**Male**	**Reference**			**Reference**			**Reference**			**Reference**			**Reference**			**Reference**		
**Female**	1.02	0.81–1.28	0.885	1.03	0.8–1.32	0.823	0.77	0.72–0.82	<0.001	0.75	0.7–0.8	<0.001	0.69	0.61–0.79	<0.001	0.62	0.54–0.71	<0.001
**Initial body weight at diagnosis**																		
**Every 10 kg increase in weight**	1.04	0.92–1.17	0.576	1.03	0.9–1.18	0.641	0.95	0.91–0.98	0.001	0.9	0.87–0.93	<0.001	0.96	0.9–1.03	0.22	0.88	0.81–0.94	<0.001
**Region**																		
**Delta region**	**Reference**			**Reference**			**Reference**			**Reference**			**Reference**			**Reference**		
**Hilly region**	0.93	0.52–1.67	0.806	0.89	0.47–1.69	0.722	1.77	1.61–1.94	<0.001	1.86	1.68–2.06	<0.001	1.51	1.26–1.82	<0.001	1.28	1.05–1.57	0.013
**Plain region**	1	0.78–1.29	0.977	0.97	0.72–1.3	0.832	0.85	0.79–0.91	<0.001	0.93	0.87–1.01	0.071	0.63	0.55–0.73	<0.001	0.6	0.52–0.69	<0.001
**Coastal region**	1.06	0.78–1.45	0.699	0.96	0.65–1.41	0.819	0.59	0.51–0.68	<0.001	0.78	0.67–0.91	0.001	0.63	0.49–0.82	0.001	0.64	0.48–0.84	0.002
**Clinical characteristics**																		
**Disease status**																		
**Pulmonary**	**Reference**			**Reference**			**Reference**			**Reference**			**Reference**			**Reference**		
**Extra-pulmonary**	1.05	0.52–2.13	0.882	1.04	0.47–2.34	0.916	1	0.83–1.2	0.944	1.01	0.83–1.22	0.932	0.8	0.52–1.22	0.297	0.65	0.42–1.01	0.058
**Sputum result**																		
**Smear positive**	**Reference**			**Reference**			**Reference**			**Reference**			**Reference**			**Reference**		
**Smear negative**	0.98	0.78–1.24	0.89	0.93	0.71–1.2	0.56	0.99	0.94–1.06	0.855	1.06	0.99–1.13	0.083	1.1	0.97–1.25	0.126	1.12	0.98–1.28	0.085
**Not done**	1.07	0.58–1.99	0.819	1.13	0.54–2.38	0.738	1.59	1.32–1.91	<0.001	1.51	1.24–1.84	<0.001	1.47	0.96–2.24	0.076	1.33	0.86–2.06	0.205
**Types of cases**																		
**New**	**Reference**			**Reference**			**Reference**			**Reference**			**Reference**			**Reference**		
**Retreated**	1.15	0.63–2.11	0.641	1.12	0.58–2.16	0.728	9.49	8.47–10.64	<0.001	7.27	6.47–8.17	<0.001	0.27	0.23–0.32	<0.001	0.27	0.23–0.32	<0.001
**Status of HIV**																		
**Negative**	**Reference**			**Reference**			**Reference**			**Reference**			**Reference**			**Reference**		
**Positive**	0.93	0.5–1.72	0.819	1	0.48–2.07	0.999	1.59	1.35–1.88	<0.001	1.66	1.4–1.96	<0.001	1.7	1.23–2.33	0.001	1.64	1.19–2.26	0.003
**Unknown**	0.99	0.79–1.24	0.937	1.01	0.79–1.3	0.933	1.85	1.73–1.97	<0.001	1.74	1.63–1.86	<0.001	1.46	1.29–1.64	<0.001	1.49	1.31–1.68	<0.001
**Support characteristics **																		
**Travel support**																		
**No**	**Reference**			**Reference**			**Reference**			**Reference**			**Reference**			**Reference**		
**Yes**	0.92	0.62–1.35	0.66	0.88	0.58–1.35	0.573	0.49	0.43–0.55	<0.001	0.6	0.53–0.68	<0.001	0.55	0.46–0.67	<0.001	0.67	0.55–0.82	<0.001
**Nutrition support**																		
**No**	**Reference**			**Reference**			**Reference**			**Reference**			**Reference**			**Reference**		
**Yes**	1.03	0.82–1.29	0.83	0.99	0.77–1.28	0.94	0.89	0.83–0.95	<0.001	1.02	0.95–1.09	0.646	0.94	0.84–1.06	0.338	1.04	0.91–1.17	0.581
**Treatment starting year**																		
**2012**	**Reference**			**Reference**			**Reference**			**Reference**			**Reference**			**Reference**		
**2013**	1.13	0.69–1.86	0.615	1.16	0.69–1.95	0.587	1.11	1.02–1.22	0.019	1.08	0.98–1.18	0.119	1.11	0.92–1.33	0.267	1.06	0.88–1.27	0.526
**2014**	1.17	0.71–1.95	0.533	1.18	0.69–2.03	0.549	1.24	1.13–1.35	<0.001	1.19	1.09–1.31	<0.001	1.27	1.07–1.52	0.008	1.22	1.02–1.46	0.032
**2015**	1.03	0.62–1.71	0.922	1.07	0.62–1.84	0.813	1.28	1.16–1.42	<0.001	1.29	1.16–1.42	<0.001	1.15	0.96–1.38	0.136	1.12	0.92–1.35	0.254
**2016**	1.2	0.72–2	0.48	1.2	0.7–2.04	0.514	1.08	0.97–1.2	0.14	1.21	1.08–1.34	0.001	1.15	0.95–1.39	0.149	1.18	0.97–1.43	0.107
**Service providers' characteristics**																		
**Age (years)**																		
**26–40**	**Reference**			**Reference**			**Reference**			**Reference**			**Reference**			**Reference**		
**40+**	1.11	0.78–1.56	0.567	1.09	0.75–1.59	0.651	0.75	0.68–0.83	<0.001	0.83	0.74–0.93	0.001	0.88	0.74–1.04	0.137	1.05	0.86–1.29	0.619
**Unknown**	0.97	0.65–1.45	0.881	0.94	0.6–1.48	0.786	1.15	1.03–1.28	0.011	1.05	0.93–1.18	0.426	1.15	0.94–1.41	0.174	1.21	0.98–1.49	0.081
**Sex**																		
**Male**	**Reference**			**Reference**			**Reference**			**Reference**			**Reference**			**Reference**		
**Female**	0.99	0.74–1.31	0.933	0.98	0.7–1.36	0.889	1.17	1.09–1.25	<0.001	1.14	1.06–1.22	<0.001	1.17	1.03–1.32	0.012	1.08	0.95–1.24	0.227
**Health facility**	0.99	0.25–3.99	0.992	1.11	0.22–5.56	0.903	1.14	0.43–3.05	0.789	0.91	0.34–2.44	0.844	0.75	0.11–5.34	0.775	0.43	0.06–3.08	0.4
**Experience with TB program (years)**																		
**0–5**	**Reference**			**Reference**			**Reference**			**Reference**			**Reference**			**Reference**		
**6–10**	1.14	0.79–1.66	0.483	1.08	0.71–1.64	0.719	0.66	0.59–0.73	<0.001	0.89	0.8–1	0.058	0.69	0.58–0.83	<0.001	0.78	0.64–0.96	0.017
**10+**	1.2	0.81–1.77	0.357	1.12	0.73–1.72	0.617	0.64	0.58–0.71	<0.001	0.89	0.79–1	0.049	0.67	0.56–0.81	<0.001	0.84	0.68–1.04	0.115

*Univariate Poisson regression model;

**Multivariate Poisson regression model; IRR: Incidence Rate Ratio; CI: Confidence Interval.

^a^Age range was from 15 to 97 years.

#### Treatment seeking channels

During the continuation phase (up to six months), compared to the walk-in patients, the patients from IPC channel were 73% more likely to be lost to follow-up, with adjusted IRR 1.73 (95% CI 1.59–1.88), whereas the patients from CHSP channel were 30% less likely to do so, with adjusted IRR 0.70 (95% CI 0.61–0.81). During continuation phase beyond six months, the differences became less marked, with adjusted IRR 1.29 (95% CI 1.09–1.53) and 0.8 (95% CI 0.64–0.99) respectively.

#### Patients’ characteristics

With every 10-year increase in patients’ age, the likelihood of loss to follow-up increased by 10% (adjusted IRR 1.10, 95% CI 1.08–1.12) during the continuation phase up to six months. However, the relationship became reversed during the continuation phase beyond six months (adjusted IRR 0.94, 95% CI 0.91–0.98). Female patients were 25% to 38% less likely to be lost to follow-up compared to males during the continuation phases with adjusted IRR 0.75 (95% CI 0.70–0.80) and 0.62 (95% CI 0.54–0.71) respectively. With every 10 kg increase in the initial body weight of patients, the likelihood of loss to follow-up became 10% to 12% lower in the continuing phases, with adjusted IRR 0.90 (95% CI 0.87–0.93), and 0.88 (95% CI 0.81–0.94) respectively. Geographically, compared to the patients from delta regions, those from hilly regions were up to 86% more likely to be lost to follow-up in the continuation phase up to six months, with adjusted IRR 1.86 (95% CI 1.68–2.06), whereas those from plain regions and coastal regions were up to 40% less likely to be lost to follow-up, especially in the continuation phase beyond six months, with adjusted IRR 0.60 (95% CI 0.52–0.69) and 0.64 (95% CI 0.48–0.84) respectively.

Compared to sputum smear-positive patients, those with no sputum results were 51% more likely to be lost to follow-up during the continuation phase up to six months, with adjusted IRR 1.51 (95% CI 1.24–1.84). But, such association were not seen in the continuation phase beyond six months, with adjusted IRR 1.33 (95% CI 0.86–2.06). Compared to the newly treated cases, retreatment cases were up to 7 times more likely to be lost to follow-up during the continuation phase up to six months with adjusted IRR 7.27 (95% CI 6.47–8.17). However, the association became reversed during the continuation phase beyond six months with adjusted IRR 0.27 (95% CI 0.23–0.32). Compared to known HIV negative patients, those with known HIV coinfection or unknown HIV status were up to 66% and 74% more likely to be lost to follow-up during the continuation phase, with adjusted IRR 1.66 (95% CI 1.4–1.96) and 1.74 (95% CI 1.63–1.86) respectively.

#### Program perspectives

The patients who received travel support were up to 40% less likely to be lost to follow-up, compared to those who did not, during the continuation phases with adjusted IRR 0.60 (95% CI 0.53–0.68). However, no significant difference in IRR was seen among patients with or without nutrition support. Chronologically, compared to year 2012, the patients registered in later years were more likely to be lost to follow-up, especially during the continuation phase up to six months, with the highest rates in 2015 (adjusted IRR 1.29, 95% CI 1.16–1.42).

During the continuation phase up to six months, the patients taking treatment from the providers over 40 years of age were up to 17% less likely to be lost to follow-up with adjusted IRR 0.83 (95% CI 0.74–0.93). In contrast, compared to the patients from the male providers, those from the female providers were 14% more likely to be lost to follow-up with adjusted IRR 1.14 (95% CI 1.06–1.22). However, these associations did not occur in the continuation phase beyond six months. There was no association between providers’ experience in TB program and loss to follow-up rates, except in one group (6–10 years of experience, beyond six months of treatment) with adjusted IRR 0.78 (95% CI 0.64–0.96).

## Discussion

The cumulative incidence of loss to follow-up among the adult TB patients differed substantially across the different treatment seeking channels, after adjustments for all available influencing factors. The overall cumulative incidence of loss to follow-up was the highest among the patients referred from IPC channel, followed by that among the walk-in patients, and that among the patients from CHSP channel. Compared to the walk-in patients, those from IPC channel were up to 73% more likely to be loss to follow-up during the continuation phase of treatment after controlling for other covariates, whereas those from CHSP channel were 30% less likely to do so.

One probable explanation such differential loss to follow-up rate across the treatment seeking channels was the difference in implementation approaches of each channel. Although the initial activities of the IPC and CHSP channels were almost the same for actively seeking presumptive TB cases, assisting in diagnosis for TB and then referring the confirmed cases to the SQHC; the IPC did not provide any support to the patients once they registered at the clinic while the CHSP continued to follow-up the patients they referred even after registered at the clinic. In addition to that, the CHSP were volunteer-based and mainly resided in rural areas, and almost all of their notified patients were in their vicinity; whereas the IPC were the recruited staff for urban slum population who may not often reside in their vicinity. Walk-in patients were passively notified by the SQHC doctors and most of the confirmed cases lived in the vicinity of clinics. Moreover, the patients tended to have close relationship with the doctors. Therefore, treatment adherence could be assured by the providers although they could not make door-to-door follow-up to the patients.

This finding pointed out that active TB case finding and notification only was not sufficient to hold the patients till the end of treatment. Increased number of TB patients put on anti-TB treatment with poor treatment outcome and high loss to follow-up are dangerous for the emergence of drug resistance tuberculosis. Our data showed that different treatment interventions with continuous TB patient care throughout the treatment period could significantly improve the loss to follow-up rate.

We also found that the loss to follow-up rates varied across the different phases of the treatment. As one would expect, the loss to follow-up rates were quite low during the first two months of the treatment (intensive phase), and rose sharply during the continuation phase up to six months, as shown in other previous studies [6, 8, 17–20]. However, the loss to follow-up rates plateaued at the continuation phase beyond 6 months. Such pattern of loss to follow-up rates were observed across all three treatment seeking channels, albeit with different baseline rates. These findings suggested that the implementers may need to pay more attention to the continuation phase, especially the first six months.

In addition to treatment seeking channels, we also found that multiple factors influenced loss to follow-up for the adult TB cases. Interestingly, these factors varied by the treatment phase as mentioned above. During the continuation phase up to 6 month, we verified some of the already known risk factors for loss to follow-up in the previous studies including: patients’ age; male patients; co-infection with HIV; and retreated cases [6–13]. This study additionally identified other probable risk factors: patients’ residency in hilly regions; unknown smear status; unknown HIV status; and possibly, patients taking treatment from relative younger providers and female providers. Of these factors, retreatment was the most significant factor, with such patients seven times more likely to be loss to follow-up, compared to the newly identified cases.

However, some of the previously identified risk factors like smear-negative pulmonary TB and extra-pulmonary TB were not significantly associated with loss to follow-up in this study. In addition, we found that patients with higher initial body weight, and patients who received travel support were less likely to be loss to follow-up. In contrary, we found that nutritional support did not make significant difference to loss to follow-up rates.

During the continuation phase beyond six months, there were fewer risk factors associated with higher loss to follow-up rates, including male patients, hilly residents, co-infection with HIV, unknown HIV status. However, the associations of increase in patients’ age and retreatment cases were reversed, and became less likely to be lost to follow-up. As in the continuation phase up to six months, patients with higher initial body weight and patients with travel support were less likely to be lost to follow-up.

There were some limitations to this study. Firstly, as we extracted the data from the client registry, the availability of the data was limited within the range of clinical records. Thus, it was possible that some information and factors associated with the major outcome of this study might be left to be examined, for instance, patients’ employment status, alcohol use, and availability of contact person. Also, the accuracy of the records was totally dependent on the clinic registers. However, we had checked the extracted data for completeness, outliers, and inconsistency, to ensure reasonable data quality. Second, as we excluded patients with other outcomes (died, failure, moved to second line and transferred out to other clinics), our estimates for cumulative incidence for loss to follow-up were relatively higher than the nominal rates from other alternative calculations. However, excluding these outcomes enabled us to dive deeper into loss to follow-up rates and to make a clean comparison against treatment success.

Despite of these limitations, we were able to generate robust estimates for loss to follow-up and the influencing factors from the biggest private sector clinics database in Myanmar. To our knowledge, this was the first study in Myanmar to demonstrate that difference in implementation approaches could actually result in significant difference in loss to follow-up rates and may possibly affect final outcomes in TB treatment.

We demonstrated that loss to follow-up rates among adult TB patients were significantly different across patients’ initial treatment seeking channels. In addition, our study shown that loss to follow-up cases mainly occurred during the continuation phase up to six months, and identified the factors influencing it. While we reconfirmed previous findings from other studies, we found that unknown statuses could be pointers to higher risk (unknown sputum results, unknown HIV status). We also found that travel support could help reducing loss to follow-up, and older service providers may also do. Based our findings, we recommend that implementation strategies for active case finding for TB should carefully consider retention strategies in parallel, and the risk-factors identified herein for loss to follow-up should be taken into account during such consideration.

## Abbreviations

CHSP: Community Health Services Provider
IPC: Interpersonal Communicator
IRR: Incidence Rate Ratio
PSI: Population Services International
SQHC: Sun Quality Health Clinics
